# Evidence for Sexual Dimorphism in the Response to TLR3 Activation in the Developing Neonatal Mouse Brain: A Pilot Study

**DOI:** 10.3389/fphys.2019.00306

**Published:** 2019-03-26

**Authors:** Raul Chavez-Valdez, Amin Mottahedin, Linnea Stridh, Tracylyn R. Yellowhair, Lauren L. Jantzie, Frances J. Northington, Carina Mallard

**Affiliations:** ^1^Division of Neonatal-Perinatal Medicine, Department of Pediatrics, Johns Hopkins School of Medicine, Baltimore, MD, United States; ^2^Institute of Neuroscience and Physiology, Sahlgrenska Academy, University of Gothenburg, Gothenburg, Sweden; ^3^Department of Pediatrics and Department of Neurosciences, The University of New Mexico, Albuquerque, NM, United States; ^4^Henan Key Laboratory of Child Brain Injury, The Third Affiliated Hospital of Zhengzhou University, Zhengzhou, China

**Keywords:** poly I:C, inflammation, caspase, cytokines, astroglia, microglia, endoplasmic reticulum stress

## Abstract

Toll-like receptor (TLR)3 activation during the neonatal period produces responses linked to the origins of neuropsychiatric disorders. Although there is sexual dimorphism in neuropsychiatric disorders, it is unknown if brain responses to TLR3 activation are sex-specific. We hypothesized that poly I:C in a post-natal day (P)8 model induces a sexually dimorphic inflammatory responses. C57BL6 mice received intraperitoneal injection of poly I:C (10 mg/kg) or vehicle [normal saline (NS)] at P8. Pups were killed at 6 or 14 h for caspase 3 and 8 activity assays, NFkB ELISA, IRF3, AP1, and GFAP western blotting and cytokines/chemokines gene expression real time qRT-PCR (4–6/group). A second group of pups were killed at 24 h (P9) or 7 days (P15) after poly I:C to assess astrocytic (GFAP) and microglia (Iba1) activation in the hippocampus, thalamus and cortex using immunohistochemistry, and gene and protein expression of cytokines/chemokines using real time RT-PCR and MSD, respectively (4–6/group). Non-parametric analysis was applied. Six hours after poly I:C, caspase-3 and -8 activities in cytosolic fractions were 1.6 and 2.8-fold higher in poly I:C-treated than in NS-treated female mice, respectively, while gene expressions of pro-inflammatory cytokines were upregulated in both sexes. After poly I:C, IRF3 nuclear translocation occurred earlier (6 h) in female mice and later (14 h) in male mice. At 14 h after poly I:C, only male mice also had increased nuclear NFκB levels (88%, *p* < 0.001) and GFAP expression coinciding with persistent IL-6 and FAS gene upregulation (110 and 77%, respectively; *p* < 0.001) and IL-10 gene downregulation (-42%, *p* < 0.05). At 24 h after poly I:C, IL-1β, CXCL-10, TNF-α, and MCP-1 were similarly increased in both sexes but at 7 days after exposure, CXCL-10 and INFγ were increased and IL-10 was decreased only in female mice. Accordingly, microglial activation persisted at 7 days after poly I:C in the hippocampus, thalamus and cortex of female mice. This preliminary study suggests that TLR3 activation may produce in the developing neonatal mouse brain a sexually dimorphic response with early activation of caspase-dependent pathways in female mice, activation of inflammatory cascades in both sexes, which then persists in female mice. Further well-powered studies are essential to confirm these sex-specific findings.

## Introduction

Toll-like receptors (TLRs) are a family of innate immune system receptors that react to both microbial stimulation and to molecules released upon tissue injury. TLR 3 modulates neuronal proliferation, differentiation, and axonal growth but also cell death in the developing brain (Cameron et al., 2007; Lathia et al., 2008; Sun et al., 2011). Neurons and glia cells express a broad variety of intracellular TLRs, including TLR3 in astrocytes and microglia (Bsibsi et al., 2002). In post-mortem specimens of preterm infants suffering periventricular white matter injury secondary to infectious or non-infectious inflammation, perinuclear TLR3 expression predominates in neurons and astrocytes (Vontell et al., 2013). Additionally, TLR3 activation increases the vulnerability to perinatal brain injury in neonatal mice (Stridh et al., 2013). In pathological conditions, TLR3 is activated by viral double stranded RNA or by endogenous ligands such as danger-associated molecular patterns (DAMPs) released from dying cells (Beg, 2002; Kariko et al., 2004). Following TLR3 activation, neuronal death may proceed via: (i) apoptosis with release of apoptotic bodies containing DNA fragments, or (ii) necrosis (or necrosis-like) with release of free suspended dead cell materials rich in DAMPs (Blasius and Beutler, 2010). In the injured developing brain, activated astrocytes and microglia engulf DAMPs forming intracellular vesicles, which fuse with TLR3-cointaing acidic endosomes (Rossi and Volterra, 2009; Ransohoff and Brown, 2012; Wakida et al., 2018). In astrocytes, TLR3 activation initiates a cytosolic TRIF-mediated cascade leading to translocation of NFκB, IRF3 and/or AP-1 to the nucleus upregulating transcription of pro-inflammatory mediators, which may perpetuate microglia activation and extend neuronal injury (Fitzgerald et al., 2003; Pobezinskaya et al., 2008).

Like most intracellular TLRs, TLR3 is a resident of the endoplasmic reticulum (ER) and traffics after uncoupling from chaperone proteins (e.g., GRP94) to the endosomal membrane (Blasius and Beutler, 2010). While chaperone proteins are essential for proper folding of TLR3 (Randow and Seed, 2001; Yang et al., 2007), other ER resident proteins are needed for translocation to the endosome (Tabeta et al., 2006). Consequently, persistent ER stress may modulate the response to many intracellular TLR ligands, including the TLR3 ligand polyinosine-polycytidylic acid (poly I:C) (Randow and Seed, 2001; Tabeta et al., 2006; Yang et al., 2007).

Activation of intracellular TLRs early in life is proposed as a mechanism leading to many neurodevelopmental and neuropsychiatric disorders (Brown, 2006; Arroyo et al., 2011; Kneeland and Fatemi, 2013), such as autism and schizophrenia (Fatemi et al., 1999). Although there is a significant male predominance in these disorders (McGrath, 2006; Loomes et al., 2017), sex differences in the mechanisms of TLR3-mediated brain injury early in life has not been reported. Since other models of neonatal brain injury suggest a greater proclivity to apoptosis in female mice and to necrosis-like cell death in male mice (Hagberg et al., 2004; Zhu et al., 2004, 2007; Northington et al., 2011; Chavez-Valdez et al., 2012; Chavez-Valdez et al., 2014), we hypothesized that poly I:C in the P8 mouse model will also induce greater caspase activation in female mice, and more prominent pro-inflammatory profile, astrogliosis, and injury in male mice. Additionally, we also studied the potential role of ER stress in sex-specific responses to TLR3 activation.

## Materials and Methods

### Mice

Experiments were performed with approval by the Ethical Committee of the Sahlgrenska Academy, University of Gothenburg, Gothenburg, Sweden (No. 18-2015; 663/17) where the handling of animals was carried out. Handling was in accordance with the National Institute of Health Guide for the Care and Use of Laboratory Animals (NIH Publications No. 80-23) and the European Convention for the Protection of Vertebrate Animals Used for Experimental and Other Scientific Purposes, Council of Europe (ETS 123). All efforts were made to minimize the number of animals used. Both male and female mice were used for these experiments.

Wild type C57BL6J mice (The Jackson Laboratory, Scanbur, Denmark) were kept in a 12 h light/dark cycle at the animal facility at University of Gothenburg (Gothenburg, Sweden). Culling was performed in the last litter to equalize the sample size for each treatment group. In total 92 (48 male and 48 female) pups were used for these experiments. Litter size varied from 6 to 10 pups. At P8, pups were assigned to either sex group based on visual inspection of external characteristics. Animal received food and water *ad libitum*. Pups were weighed at P8, P9, and P15 to evaluate differences in growth by group. At P8 mice received intraperitoneal (IP) injection of the TLR3 agonist poly I:C (Poly I:C- Low Molecular Weight; InvivoGen, Toulouse, France) reconstituted in LPS-free normal saline (NS) to 1 mg/mL and injected at 10 mg/kg or vehicle (LPS-free NS). Pups were killed at 6 or 14 h for: (i) caspase 3 and 8 activity assays; (ii) NFkB ELISA; (iii) western blot for IRF3, AP1, and GFAP; and (iv) pro-inflammatory (IL-1β, TNFα, FAS, CXCL-1, CXCL-10, MCP-1, INF-β, INF-γ, and IL-6) and anti-inflammatory (IL-10) cytokines/chemokines gene expression by real time qRT-PCR. A second group of pups were killed at 24 h (P9) or 7 days (P15) after poly I:C exposure to evaluate: (i) microscopic injury using Nissl counterstaining; (ii) astroglia and microglia activation using GFAP and Iba1 IHC, respectively; and (iii) gene and protein expression of a similar battery of cytokines/chemokines as described above, by real-time RT-PCR and multiplex electrochemiluminescent immunoassay, respectively. All samples were coded and run by laboratory personnel blinded to treatments, sex, and times.

In all cases, mice were anesthetized and perfused intracardially with NS prior to brain dissection. Brains were snap frozen, and stored at -80°C until analysis except for the hemispheres used for histological evaluation, which were fixed in 4% paraformaldehyde (PFA) by immersion for 1 week followed by cryoprotection using 15 and 30% sucrose gradient prior to freezing at -80°C.

### Caspase 3 and 8 Activity Assays

Cytoplasmic and nuclear fractions were prepared using a piece of brain from the same brains used for RNA isolation using a Subcellular Protein Fractionation Kit (Thermo Scientific, Rockford, IL, United States). Buffers were part of the kit and supplemented with protease and phosphatase inhibitors. Approximately 150 mg of tissue washed in ice-cold phosphate-buffered saline (PBS, pH 7.2) was homogenized in Cytoplasmic Extraction Buffer at 1:10 (w:v) using a standard tissue grinder and transferred to a tissue strainer. Following centrifugation at 500 × *g* for 5 min, supernatant (cytoplasmic fraction) was used for Caspase 3 and 8 activity assays. The pellet was sequentially resuspended in ice cold Membrane and Nuclear Extraction Buffers at 1:6.5 and 1:2.25 (w:v), respectively. The final supernatant, the nuclear fractions, were stored at -80°C for nuclear factor κB (NFκB) measurement. Twenty percent (w/v) glycerol was added to the fractions and protein concentrations were determined using the Bradford assay (Bradford, 1976). Cross-contamination between nuclear and cytosolic fractions was minimal following immunoblotting for Lamin B1 (nuclear protein) and HSP90 (cytosolic protein), details below. Cytosolic fractions were assayed for caspase 3 or caspase 8 activity using the respective Colorimetric Activity Assay Kits, (APT165 and APT129; Millipore, Billerica, MA, United States). Cytosolic fractions (400 μg) were plated in a 96-well culture plate and reconstituted in cell lysis buffer and diluted (1:1 v:v) in assay buffer provided by the manufacturer prior to incubation for 60 min at 37°C with the substrate Ac-DEVD-pNA (N-Acetyl-Asp-Glu-Val-Asp-*p-*nitroanilide) for caspase 3 or Ac-IETD- pNA (*N*-acetyl-Ile-Glu-Thr-Asp-ρ-nitroaniline) for caspase 8 activity assay. The chromophore *ρ*-NA was measured at 405 nm using a Model 680 Microplate Reader (Bio-Rad, Hercules, CA, United States). Semiquantitative measurement of ρNA concentrations in samples was determined indirectly using the linear model of absorbance.

### Nuclear Factor-κB Transcription Factor Determination Using ELISA

Nuclear fractions were used to determine concentrations of NF*κ*B bound to the consensus sequence 5′-GGGACTTTCC-3′ in the biotinylated capture probe and detected by exposure to the primary rabbit anti-NF*κ*B p65 antibody (1:1000) followed by a horseradish peroxidase-conjugated goat anti-rabbit secondary detection antibody (1:500) using the EZ-TFA Transcription Factor Assay (Millipore, Temecula, CA, United States). Positive (TNF*α*-treated HeLa whole-cell extract), specific competitor (NF*κ*B competitor oligonucleotide), and negative controls were used to establish specificity and sensitivity at λ450/ 650 nm using a microplate reader.

### Western Blot for IRF3, AP1, GFAP, GRP94

Twenty μg-aliquots of cytosolic (for all protein targets) or nuclear fractions (for IRF3 and AP1) were diluted 3:1 (v:v) in 4X loading buffer under reducing conditions. Samples were loaded in 4–20% mini-protean TGX polyacrylamide precast protein gels (Biorad, Inc., Hercules, CA, United States) and transferred to nitrocellulose membrane using TransBlot Turbo Midi-size (Biorad, Inc.). Blots were stained with Ponceau S and blocked using 2.5% BSA with 0.1% Tween-20 in 50 mM Tris buffered saline (TBST, pH 7.4) before incubation overnight at 4°C in primary antibodies at 1:1000 (GFAP), and 1:500 (IRF3, AP1, and GRP94). After exposure blots were washed with TBST, exposed to secondary antibodies for 1 h and then developed using enhanced chemiluminescence (Clarity Western ECL Substrate, Biorad, Inc.). Loading control at 1:5000 was performed using β-actin for cytosolic fractions and histone H1.4 (both form Sigma). To quantify protein immunoreactivity, optical density (OD) was determined with ImageJ adjusted for background.

### Antibodies

IRF3 (Proteintech Group, Inc. 11312-1-AP: RRID: AB_2127004): rabbit polyclonal IgG antibody raised against the IRF3 fusion protein Ag1858 detecting a single band at 49 to 55 kDa (2 μg/ml). AP1 (Proteintech Group, Inc. 22114-1-AP: RRID: AB_2750860): rabbit polyclonal IgG antibody raised against the p39 fusion protein Ag17419 detecting a single band at 40 to 46 kDa (2 μg/ml). GFAP (Proteintech Group, Inc. 16825-1-AP: RRID: AB_ 2109646): rabbit polyclonal IgG antibody raised against the GFAP fusion protein Ag10423 detecting a single band at 45 to 50 kDa (1 μg/ml). GRP94 (Proteintech Group, Inc. 60012-1-Ig: RRID: AB_2119056): mouse monoclonal IgM antibody raised against the GRP94 fusion protein Ag1439 detecting a single band at 95 kDa (2 μg/ml). For evaluation of nuclear-cytosol cross-contamination of the fractions, the following antibodies were used: (i) HSP90 (cytosolic protein, Cell Signaling Technology, Inc. 4875: RRID: AB_2233331): rabbit polyclonal antibody raised against the synthetic peptide surrounding Glu289 of the HSP protein of human origin detecting a single band at 90 kDa without cross-reactivity with other heat-shock proteins (1 μg/ml); and (ii) Lamin B1 (nuclear protein, Cell Signaling Technology, Inc. 13435: RRID: AB_2737428): rabbit monoclonal antibody produced by immunization with a synthetic peptide corresponding to residues surrounding Lys415 of lamin B1 protein of human origin detecting a band at 68 kDa (1 μg/ml).

### Gene Expression Using Real Time qRT-PCR

Total RNA was extracted from two combined pieces from anterior and posterior brain from mice pups treated as described above. Tissues were obtained at 6, 14, 24 h (P9) and 7 days (P15) after normal saline or poly I:C exposure. PureLink^TM^ RNA mini kit purification system (Invitrogen, Carlsbad, CA, United States) was used for RNA extraction and measured using a spectrophotometer at 260 nm absorbance. Total RNA (1 μg) was used for generation of complementary single strand DNA (cDNA) using iScript cDNA synthesis kit (BioRad). Reverse transcription protocol included 5 min at 25°C; 30 min at 42°C and 5 min at 85°C. cDNA was then used for gene amplification. The following SYBR Green Based primers from Qiagen (Germantown, MD, United States) were used: IL-6 (QT00098875), IL-1β (QT01048355), TNF-α (QT00104006), Fas (QT00095333), IFN-β (QT00249662), INF-γ (QT01038821), interferon-induced protein-10 (IP-10/CXCL-10) (QT00093436), CXCL-1 (QT00115647) monocyte chemoattractant protein-1 (MCP-1) (QT00167832), IL-10 (QT00106169), including the housekeeping genes GAPDH (QT01658692), and tyrosine 3-monooxygenase/tryptophan 5-monooxygenase activation protein, zeta polypeptide (YWHAZ; QT00105350). The amplification protocol included 40 cycles of 30 s at 95.0°C, 1 min at 60°C ending with 30 s at 72.0°C. Fold difference in gene expression was then corrected by the geometric mean of GAPDH and YWHAZ gene expression using the using the Pfaffl method (Pfaffl, 2001). Melting curves confirmed amplification of single PCR products.

### Cytokine and Chemokine Measurement in Brain Crudes

To measure cytokine and chemokine concentrations in brain, a V-plex multi-plex electrochemiluminescent immunoassay platform (MesoScale Discovery, Gaithersburg, MD, United States) was used consistent with our prior studies (Maxwell et al., 2015; Robinson et al., 2016, 2018; Yellowhair et al., 2018). This system has been validated by traditional ELISA and produces measurements with high content validity and inter-assay variations less than 12%. Specifically, brain lysate (100 μg) or standard was loaded on to a 96-well plate in duplicate per manufacturer’s specification. Plates were read on a Quickplex SQ 120 Imager (Mesoscale Discovery, Gaithersburg, MD, United States).

### IHC for GFAP and Iba1

After intracardiac perfusion with NS and dissection, the brain hemispheres assigned for histological analysis were fixed by immersion in 4% PFA in 0.1 M PBS for 7 days. Tissues were cryoprotected with graded immersion in 15% and then 30% sucrose in PBS until the tissue sank, then frozen and stored in -80°C until cut at 50 μm on a freezing microtome. Sections obtained from animals killed 24 h or 7 days after normal saline or poly I:C exposure were used to assess astroglia (GFAP) and microglia (Iba1) activation and overall microscopic evidence of injury (Nissl counterstaining for GFAP IHC). Floating IHC was performed as previously described (Chavez-Valdez et al., 2018) with whole rabbit antisera anti-GFAP (DAKO/Agilent Technologies, Santa Clara, CA, United States; 1:1000), or anti-Iba1 (Wako Chemicals USA, Inc., Richmond, VA, United States; 1:500) followed by goat anti-rabbit antibody (1:200) used as the secondary antibody using DAB as the chromogen for GFAP and using Alexa Fluor 568 for immunofluorescence for Iba1. Cresyl-violet (CV) counterstaining was performed in those sections previously immunostained for GFAP to assess histological structure. GFAP IHC slides were imaged using a light microscope (Nikon Eclipse E400, Nikon, Minato, Japan), to produce high resolution photomicrographs (1344 × 1024 pixels). Percent area of GFAP immunostaining in the region of interest (hippocampal CA1 and DG, thalamic ventroposterior nuclei, and cingulate cortex) inserted within the 4 X × 20 X high-resolution photomicrographs was calculated using the color threshold function of ImageJ 1.8.0 software (NIH, Bethesda, MD, United States). Immunofluorescent images for Iba1 were captured at 512 × 512 pixels using a Laser Scanning Confocal Microscope LSM700 from Carl Zeiss AG (Oberkochen, Germany). Total Iba1 immunofluorescence signal was quantified using the histogram function in the Zen 2.3 blue edition (Carl Zeiss Microscopy GmbH, Jena, Germany).

### Antibodies

*GFAP* (DAKO Z0334; RRID:AB_10013382): rabbit polyclonal antibody raised against GFAP isolated from cow spinal cord with no reported cross reactivity (1 μg/ml). *Iba1* (WAKO 019-19741; RRID:AB_839504): rabbit polyclonal antibody raised against a synthetic peptide corresponding to the C-terminus of Iba1 purified by antigen affinity chromatography, with no reported cross-reactivity with neuronal or astrocytic markers (1 μg/ml).

### Statistical Analysis

For analysis of two related groups, such as the fold-change in gene expression relative to control (NS group) produced by the Pfaffl method, non-parametric Wilcoxon signed-rank test vs. control stratified by sex was applied. Data were presented as box and whisker plot, where the box was limited by the 25^th^ and 75^th^ percentiles and the solid line represented the median. The discontinued line sitting at 1 represented the relative expression of the comparison group (NS). For within sex and time group analysis of two independent treatments (NS and Poly I:C), Mann–Whitney *U*-test was applied. Significance was assigned by *p* ≤ 0.05 in all cases. Analysis was performed using IBM SPSS Statistics 24V (IBM Corporation, Armonk, NY, United States).

## Results

### Poly I:C Exposure and Growth

The median weight of the pups included in both treatment groups, NS and poly I:C, was similar prior to injection at P8 (*p* = 0.76 for males and *p* = 0.84 for females). By 24 h (P9) or 7 days (P15) after injection the rate of growth was 0.45 to 0.50 mg/day for pups in the NS and those in poly I:C treatment groups. No difference in growth rate by treatment group was documented in male pups (p _(24 h)_ = 0.70, p _(7 d)_ = 0.31) or female pups (p _(24 h)_ = 0.82, p _(7 d)_ = 0.18).

### Sex Differences in Caspase 3 and Caspase 8 Activity in Mice After Poly I:C

**FIGURE 1:**
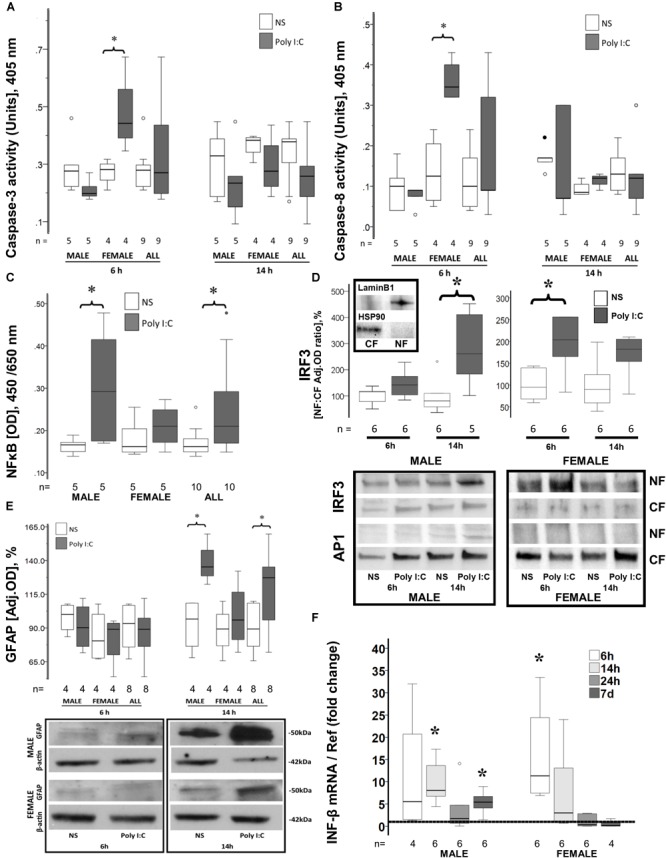
Early sex-specific responses to poly I:C in the brain of P8 mice. Increased caspase-3 **(A)** and caspase-8 activity **(B)**, IRF3 nuclear translocation **(D)**, and INF-β gene upregulation **(F)** occur 6 h after poly I:C in the brain of female mice. Increased NFκB **(C)** and IRF3 **(D)** nuclear translocation, GFAP expression **(E)** and INF-β gene upregulation **(F)** occur 14 h after poly I:C in the brain of male mice. Data are presented using box and whiskers plots, where boxes are limited by the 75^th^ and 25^th^ percentiles interquartile range (IQR)] and whiskers are limited by the last data point within 1.5 times the IQR from the median (continuous line inside the box). **(A–E)** White boxes represent NS-treated pups, while gray boxes represent poly I:C-treated pups. **(D)** Representative immunoblots showing translocation of IRF3 and AP1 from the cytosolic fraction (CF) to the nuclear fraction (NF) in males and females at 6 and 14 h after poly I:C. Top immunoblots show purity of the NF and CF using Lamin B1 and HSP90, respectively. **(E)** Representative immunoblots showing GFAP expression in cytosolic fractions. **(F)** Shows the fold change in INF-β mRNA levels in the brain of male and female mice at 6, 14, 24 h and 7 days after poly I:C relative to NS-treated mice (discontinuous line sitting at 1). °, outlier (between 1.5 and 3 times the IQR); ^•^, extreme (> 3 times the IQR); ^∗^*p* < 0.05.

Neither caspase 3 (Figure 1A), nor caspase 8 (Figure 1B) activity increased at 6 or 14 h after poly I:C exposure in male mice (*n* = 5/group). To the contrary, in female mice poly I:C increased caspase 3 activity by 57% from 0.28 (IQR 0.22–0.30) units to 0.44 (IQR 0.37–0.62) units at 6 h after exposure (*p* = 0.02 vs. NS exposed female mice, *n* = 4/group; Figure 1A). This increase in caspase-3 activity resolved at 14 h after poly I:C exposure. Accordingly, in female mice poly I:C also increased caspase-8 activity from 0.12 (IQR 0.05–0.22) units to 0.35 (0.32–0.42) units at 6 h after exposure (*p* = 0.02 vs. NS exposed female mice, *n* = 4/group; Figure 1B). Similar to caspase 3, caspase 8 increase resolved by 14 h after poly I:C exposure.

### Sex Differences in NFκB and IRF3 Nuclear Translocation in Response to Poly I:C

While no difference in nuclear NFκB was observed 6 h after poly I:C (data not shown), 14 h after poly I:C exposure, NFκB nuclear levels increased by 28% in all mice (*p* = 0.04 vs. NS-exposed, *n* = 10/group, Figure 1C). When stratified by sex, no change in nuclear NFκB levels after poly I:C exposure was documented in female mice, while an increase of 76% (*p* = 0.04 vs. NS group, *n* = 5/group, Figure 1C) was documented in male mice at 14 h after poly I:C. Other pathways downstream of TLR3 activation were also studied. Nuclear translocation of IRF3 was temporally distinct between females and males. While IRF3 translocation to the nucleus occurred 6 h after poly I:C in female mice (*p* = 0.01 vs. NS, *n* = 6/group), this change was only observed at 14 h after poly I:C in the male mice (*p* = 0.01 vs. NS, *n* = 5–6/group; Figure 1D). No significant nuclear translocation of AP1 was observed at either 6 or 14 h after poly I:C in either sex (Figure 1D).

### Increase Early Astrocytic Activation in Male Mice Exposed to Poly I:C

In activated astrocytes, TLR3 activation initiates a cytosolic cascade leading to nuclear translocation of NFκB, IRF3, and/or AP-1 and downstream transcription of pro-inflammatory mediators, which may perpetuate microglia activation and extend neuronal injury (Fitzgerald et al., 2003; Pobezinskaya et al., 2008). Expression of GFAP, an astrocytic marker, was unchanged 6 h after poly I:C exposure, while it was increased by 42.7% (*p* = 0.003 vs. NS-exposed mice, *n* = 8/group) at 14 h after exposure. Temporarily coinciding with their later nuclear translocation of NFκB and IRF3, only male mice responded with a 40% (*p* = 0.02, *n* = 4; Figure 1E) increase in GFAP expression at 14 h after poly I:C exposure, while female mice did not show any change.

### Sexual Dimorphism in the Temporal Patterns of INF-β Gene Expression After Poly I:C

INF-β is the prime final product downstream of TLR3 activation. Six hours after poly I:C exposure, INF-β gene expression was upregulated by 8.8-fold in all mice (*p* = 0.005 vs. NS, *n* = 10; data not shown). INF-β gene upregulation derived from the early upregulation in female mice (11.3-fold, *p* = 0.03, *n* = 6; Figure 1F). Fourteen hours after poly I:C the gene expression of INF-β was upregulated by sevenfold (*p* = 0.005 vs. NS, *n* = 12; data not shown) in all mice, at this time point the increase derived from the upregulation in male mice (8.1-fold, *p* = 0.03, *n* = 6; Figure 1F). This pattern temporally mirrors the sexual dimorphism in IRF3 and NFκB nuclear translocations. By 24 h after poly I:C INF-β gene expression returns to levels similar to those seen in NS-treated mice. However, 7 days after poly I:C, INF-β becomes again upregulated in male mice (5.4-fold, *p* = 0.03 vs. NS, *n* = 6; Figure 1F).

### Prolonged Pro-inflammatory Gene Profile After Poly I:C Exposure

**FIGURE 2:**
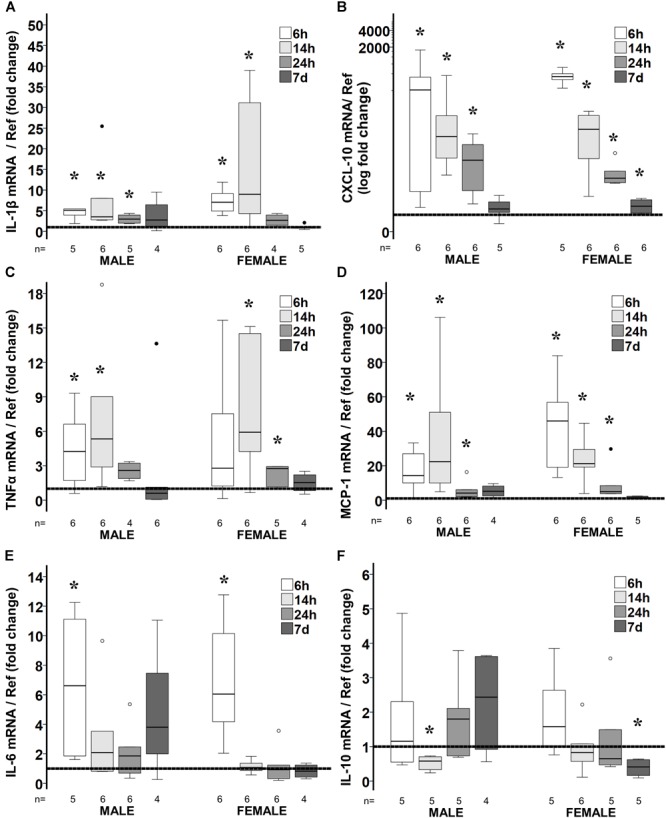
Temporal changes in brain cytokine/ chemokine gene expression in response to poly I:C exposure at P8. Fold change in gene expression of IL-1β **(A)**, CXCL-10 **(B)**, TNFα **(C)**, MCP-1 **(D)**, IL-6 **(E)**, and IL-10 **(F)** in the brain of poly I:C treated mice relative to NS-treated mice. Data are shown using box and whiskers plots, where boxes are limited by the 75^th^ and 25^th^ percentiles (IQR) and whiskers are limited by the last data point within 1.5 times the IQR from the median (continuous line inside the box). Data from male and female mice at 6, 14, 24 h and 7 days after poly I:C relative to NS-treated mice (discontinuous line sitting at 1) is shown. °, outlier (between 1.5 and 3 times the IQR); ^•^, extreme (>3 times the IQR); ^∗^*p* < 0.05.

Similar to INF-β, gene expression profile of many cytokines was sexually dimorphic following poly I:C exposure at P8. In male mice, poly I:C upregulated the mRNA level of the following pro-inflammatory genes at 6 h after exposure: IL-1β (5.1-fold, *p* = 0.04, *n* = 5; Figure 2A); CXCL-10 (345-fold *p* = 0.02, *n* = 6; Figure 2B); TNF-α (4.3-fold, *p* = 0.04, *n* = 6; Figure 2C); MCP-1 (14.3-fold, *p* = 0.04, *n* = 6; Figure 2D); FAS (twofold, *p* = 0.01, data not shown); and IL-6 (6.6-fold, *p* = 0.04, *n* = 5, Figure 2E); without changing the expression of the anti-inflammatory cytokine IL-10 (Figure 2F). Similarly, an early pro-inflammatory profile was documented 6 h after poly I:C exposure in female mice with upregulation of: IL-1β (sevenfold, *p* = 0.03, *n* = 6; Figure 2A); CXCL-10 (594-fold, *p* = 0.04, *n* = 5; Figure 2B); MCP-1 (46-fold, *p* = 0.03, *n* = 6; Figure 2D); FAS (2.8-fold, *p* = 0.04, data not shown); and IL-6 (sixfold, *p* = 0.03, *n* = 6; Figure 2E). In male mice, 14 h after poly I:C exposure all pro-inflammatory markers remained upregulated, including: IL-1β (3.5-fold, *p* = 0.02, *n* = 6; Figure 2A); CXCL-10 (51-fold, *p* = 0.02, *n* = 6; Figure 2B); TNF-α (5.3-fold, *p* = 0.02, *n* = 6, Figure 2C); MCP-1 (22-fold, *p* = 0.02, *n* = 6; Figure 2D), and FAS (1.8-fold, *p* = 0.01, *n* = 6; data not shown);but additionally IL-10 was downregulated by 42% (*p* = 0.04, *n* = 5; Figure 2F). Unlike male mice, the gene expression of several pro-inflammatory markers returned to normal in female mice at 14 h after poly I:C including, IL-6 and FAS and at 24 h after poly I:C, IL-1β. Additionally, the brains of female mice did not respond to poly I:C with down-regulation of IL-10 14 h after exposure, as documented in male mice (Figure 2F). As expected a trajectory to return to levels similar to those in NS-treated mice was observed between 24 h and 7 days after poly I:C. Thus, at 7 days after poly I:C IL-1β, TNF-α, and MCP-1 gene expressions were similar to those in NS-treated mice in both sexes. However, only in female mice, upregulation of the CXCL-10 (*p* = 0.03, *n* = 6) and downregulation of IL-10 (*p* = 0.04, *n* = 5) genes were present at 7 days after poly I:C (Figure 2B,F).

An extensive battery of pro-inflammatory cytokines/chemokine protein levels were measured in poly I:C treated mice at 24 h and 7 days after HI (Table 1). Matching the gene expression data, IL-1β, CXCL-10, TNF-α, and MCP-1 were increased at 24 h after poly I:C exposure in both sexes. Also congruent with the gene expression results, 7 days after poly I:C the levels of CXCL-10 (63%, *p* = 0.05), CXCL-1 (19%, *p* = 0.01) and INF-γ (38%, *p* = 0.006) were elevated, while the level of IL-10 (-21%, *p* = 0.05) was decreased only in female mice (Table 1).

**Table 1:** Cytokine expression following poly I:C exposure stratified by sex.

		Males			Females		
		Saline	Poly I:C	*p*-value	Saline	Poly I:C	*p*-value
**Pro-inflammatory** (*n* = 6/group) (pg/100 μg protein)							
INF-γ Median [IQR]	**24 h**	0.103 [0.08–0.12]	0.119 [0.11–0.15]	0.10	0.091 [0.08–0.09]	0.096 [0.80–0.11]	0.52
	**7 days**	0.074 [0.06–0.09)	0.063 [0.04–0.07]	0.10	**0.045 [0.03–0.05]**	**0.062 [0.06–0.07]**	**0.006^∗^**
IL-1β Median [IQR]	**24 h**	**0.311 [0.27–0.37]**	**0.545 [0.44–0.64]**	**0.006^∗^**	**0.367 [0.29–0.45]**	**0.599 [0.47–0.74]**	**0.01^∗^**
	**7 days**	0.395 [0.34–0.43]	0.286 [0.24–0.43]	0.42	0.304 [0.27–0.35]	0.301 [0.26–0.33]	0.87
IL-2 Median [IQR]	**24 h**	0.101 [0.06–0.10]	0.095 [0.07–0.10]	0.56	0.108 [0.05–0.10]	0.106 [0.08–0.24]	0.43
	**7 days**	ND	ND	–	ND	ND	–
IL-5 Median [IQR]	**24 h**	**0.466 [0.40–0.58]**	**0.605 [0.53–0.64]**	**0.05^∗^**	0.483 [0.45–0.55]	0.634 [0.47–0.71]	0.10
	**7 days**	0.359 [0.29–0.39]	0.358 [0.26–0.38]	1.00	0.349 [0.33–0.37]	0.350 [0.28–0.39]	1.00
IL-12 Median [IQR]	**24 h**	18.16 [13.9–22.6]	23.92 [18.1–31.6]	0.26	20.48 [16.8–26.4]	24.49 [17.5–29.4]	0.42
	**7 days**	14.55 [9.71–18.9]	15.25 [7.62–19.9]	0.87	15.81 [6.39–18.4]	13.49 [9.62–16.4]	0.72
CXCL-1 Median [IQR]	**24 h**	2.79 [2.65–3.06]	3.19 [2.76–3.61]	0.10	2.66 [2.33–2.77]	3.04 [2.57–3.26]	0.10
	**7 days**	1.79 [1.39–1.99]	2.14 [1.73–2.34]	0.10	**1.76 [1.57–1.96]**	**2.09 [1.98–2.35]**	**0.01^∗^**
CXCL-10 Median [IQR]	**24 h**	**13.34 [10.8–13.3]**	**691.4 [521.1–1043]**	**0.004^∗^**	**13.92 [12.4–15.2]**	**480.9 [346.7–1782]**	**0.004^∗^**
	**7 days**	10.34 [9.6–13.7]	11.85 [10.6–12.6]	0.52	**9.45 [7.21–14.7]**	**15.42 [12.6–20.5]**	**0.05^∗^**
TNF-α Median [IQR]	**24 h**	**0.243 [0.21–0.31]**	**0.402 [0.37–0.46]**	**0.004^∗^**	**0.269 [0.25–0.32]**	**0.406 [0.38–0.43]**	**0.04^∗^**
	**7 days**	0.233 [0.21–0.26]	0.217 [0.18–0.26]	0.42	0.212 [0.18–0.24]	0.176 [0.16–0.23]	0.33
MCP-1 Median [IQR]	**24 h**	**2.14 [1.92–2.46]**	**40.88 [30.9–47.6]**	**0.004^∗^**	**1.819 [1.72–2.19]**	**38.58 [30.3–62.5]**	**0.004^∗^**
	**7 days**	1.546 [1.47–1.76]	1.662 [1.52–1.85]	0.42	1.509 [1.23–1.84]	1.706 [1.54–1.89]	0.10
**Modulatory** (*n* = 6/group) (pg/100 μg protein)							
IL-6 Median [IQR]	**24 h**	8.72 [8.05–10.5]	10.52 [9.21–10.8]	0.20	8.46 [7.91–10.4]	10.35 [8.53–11.4]	0.20
	**7 days**	7.62 [6.87–8.44]	7.54 [5.79–8.23]	0.75	7.02 [6.54–7.42]	7.26 [5.68–7.61]	1.00
**Anti-inflammatory** (*n* = 6/group) (pg/100 μg protein)							
IL-4 Median [IQR]	**24 h**	0.385 [0.23–0.66]	0.343 [0.28–0.39]	0.52	0.253 [0.18–0.42]	0.266 [0.20–0.36]	0.87
	**7 days**	0.149 [0.08–0.24]	0.139 [0.13–0.20]	0.87	0.148 [0.08–0.25]	0.132 [0.09–0.18]	0.75
IL-10 Median [IQR]	**24 h**	5.44 [4.52–6.26]	5.07 [4.42–5.91]	0.74	6.55 [5.64–7.09]	6.11 [5.75–6.74]	0.52
	**7 days**	4.64 [4.12–5.34]	4.63 [4.13–5.61]	0.87	**5.24 [5.05–5.42]**	**4.13 [3.32–5.17]**	**0.05^∗^**

*Bold font signifies statistical significance.*

### Astrocyte and Microglia Activation Following Poly I:C

**FIGURE 3:**
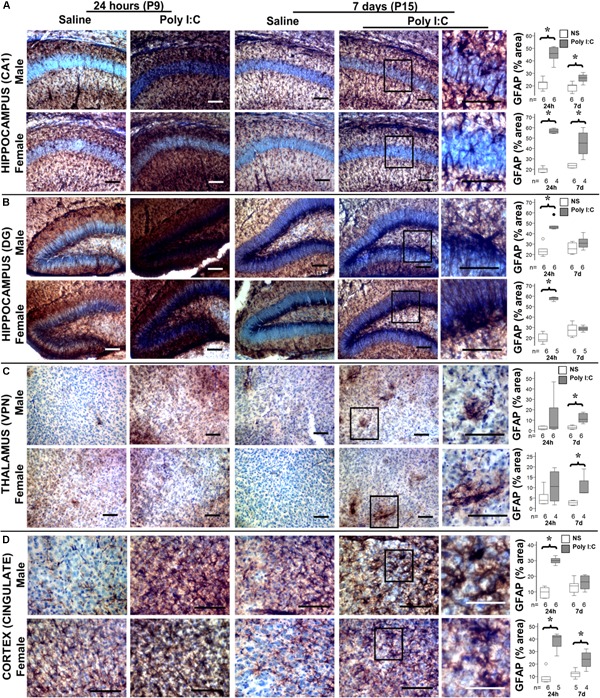
Temporal changes in astrocytic activation after poly I:C exposure at P8. Representative photomicrographs of GFAP immunoreactivity in the CA1 **(A)** and dentate gyrus (DG, **B**) subfields of the hippocampus, ventroposterior nuclei (VPN) of the thalamus **(C)**, and cingulate cortex **(D)** of male and female mice at 24 h and 7 days after treated with normal saline or poly I:C. High magnification details are provided for 7 days after poly I:C. In all panels, bar represents 100 μm, with the exception of the detail higher magnification panel for the cingulate cortex at 7 days after poly I:C, where bar represent 50 μm. Semi-quantitative analyses of percent area of GFAP immunostaining per sex and region of interest are shown using box and whiskers plots, where boxes are limited by the 75^th^ and 25^th^ percentiles (IQR) and whiskers are limited by the last data point within 1.5 times the IQR from the median (continuous line inside the box). Data from male (top) and female (bottom) mice at 24 h and 7 days after treatment with NS (white boxes) or poly I:C (gray boxes) are shown. °, outlier (between 1.5 and 3 times the IQR); ^•^, extreme (>3 times the IQR); ^∗^*p* < 0.05 (*n* = 6/ group). Twenty-four hours after poly I:C, significant astrocytic activation was observed in the hippocampus (CA1 and DG subfields) and cingulate cortex. At 7 days after poly I:C, astrocytic activation greatly diminished with the exception of the VPN of the thalamus, demonstrating greater evidence of astrocytosis at 7 days than at 24 h after poly I:C. No sex-specific differences were observed. Detailed information about percent area of GFAP immunoreactivity in each area of interest is provided in Table 2.

**FIGURE 4:**
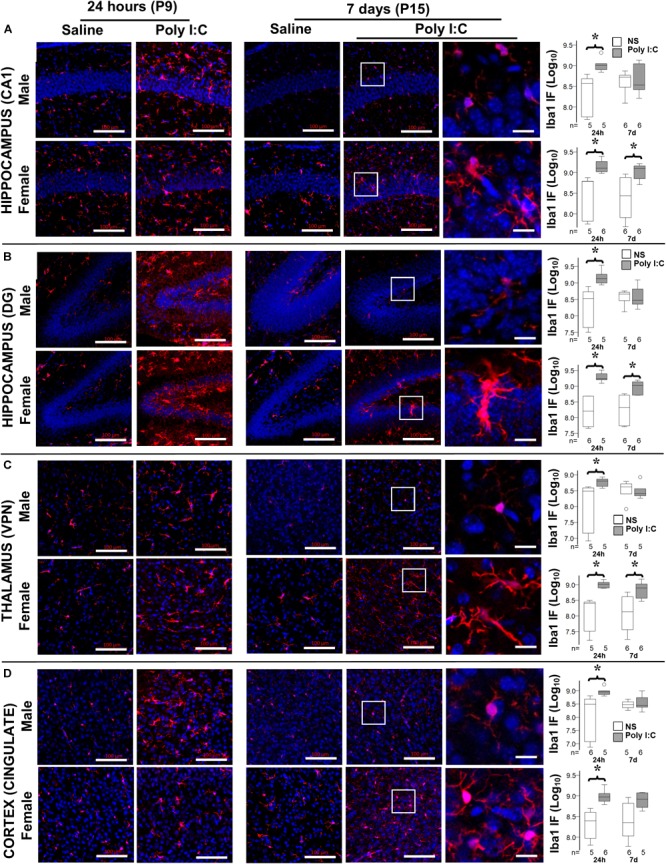
Temporal changes in microglia activation after poly I:C exposure at P8. Representative photomicrographs of Iba1 immunofluorescence in the CA1 **(A)** and dentate gyrus (DG, **B**) subfields of the hippocampus, VPN of the thalamus **(C)**, and cingulate cortex **(D)** of male (top row) and female mice (bottom row) at 24 h and 7 days after treatment with normal saline (NS) or poly I:C. High magnification datails show at 7 d after poly I:C. In all panels, bar represents 100 μm, with the exception of the detail high magnification panels for 7 days after poly I:C, where bar represent 20 μm. Semi-quantitative analyses of Iba1 immunofluorescence per sex and region of interest are shown using box and whiskers plots, where boxes are limited by the 75^th^ and 25^th^ percentiles (IQR) and whiskers are limited by the last data point within 1.5 times the IQR from the median (continuous line inside the box). Data from male (top) and female (bottom) mice at 24 h and 7 days after treatment with NS (white boxes) or poly I:C (gray boxes) are shown. Data are presented as the decimal logarithmic transformation of the total immunofluorescence signal in arbitrary units. °, outlier (between 1.5 and 3 times the IQR); ^•^, extreme (>3 times the IQR); ^∗^*p* < 0.05 (*n* = 4–6/group). Twenty-four hours after poly I:C, significant microglia activation was observed in all regions in both sexes. At 7 days after poly I:C, microglia activation significantly diminished in male mice (top), while persisted in all brain regions in female mice (bottom). Detailed information about immunofluorescence provided in Table 3.

Although by 14 h after poly I:C GFAP protein level was increased only in male mice, at 24 h after poly I:C GFAP expression was equally observed in the hippocampus (CA1 and dentate gyrus subfields) and cingulate cortex of both male and female mice (Figure 3 and Table 2). Seven days after poly I:C, evidence of astrocytic activation greatly diminished in both sexes in all regions (Figure 3A,B,D) with the exception of the ventroposterior nuclei of the thalamus, demonstrating greater number of reactive astrocytes at 7 days than at 24 h after poly I:C (Figure 3C and Table 2). Similar to GFAP staining, Iba-1 staining peaked at 24 h after poly I:C in the hippocampus and the cingulate cortex (Figure 4) but unlike GFAP-staining, residual Iba-1 immunofluorescence at 7 days after exposure show a sex specific pattern. While evidence of microglia activation was almost resolved in the hippocampus (Figure 4A [CA1] and 4B [DG]), thalamus (Figure 4C) and cingulate cortex (Figure 4D) at 7 days after poly I:C in male mice; significant residual activation was present in all those brain regions in female mice (Figure 4). Semi-quantitative analysis of Iba1 immunofluorescence in those regions are shown in Figure 4 and Table 3.

**Table 2:** Percent area of GFAP immunostaining per region.

		24 h (P9)			7 days (P15)		
Region	Sex	Saline	Poly I:C	*p*-Value	Saline	Poly I:C	*p*-Value
Hippocampus CA1 Median % [IQR]	**Male**	22.6 [18.9–22.9]	45.9 [42.9–49.5]	**0.002**	20.3 [16.8–20.7]	26.4 [24–28.4]	**0.009**
	**Female**	19.7 [17.6–20.7]	56.5 [54.9–58.4]	**0.01**	23.6 [21.8–25.2]	45.2 [37.8–52.4]	**0.01**
Hippocampus DG Median % [IQR]	**Male**	22.6 [20.5–24.7]	46.2 [44.9–47.4]	**0.002**	24.4 [21.6–30.2]	30.8 [27.9–33.9]	NS
	**Female**	18.2 [16.6–22.8]	58.3 [57.1–58.8]	**0.004**	27.3 [22.9–31.1]	28.7 [27.1–30.5]	NS
Thalamus VPN Median % [IQR]	**Male**	1.8 [1–3.9]	3.3 [1.9–17.5]	NS	2.8 [1.9–4.2]	10.8 [8.7–15.2]	**0.002**
	**Female**	3.9 [2.4–6.3]	10.6 [4.5–16.7]	NS	2.7 [1.9–3.5]	7.6 [7.5–10.5]	**0.01**
Cingulate Cortex Median % [IQR]	**Male**	9.9 [6.8–12.2]	29.9 [28.9–31.1]	**0.002**	13.8 [10.6–15.1]	13.8 [10.6–15.1]	NS
	**Female**	7.5 [6.3–9.3]	41.7 [34.1–42.9]	**<0.001**	12.4 [9.9–13.6]	23.9 [19.9–27.4]	**0.003**

*Data show as percent (%) area of GFAP immunoreactivity in the region of interest within high magnification photomicrograph. *n* = 4–6/group. Mann–Whitney *U-*test used for analysis. DG, dentate gyrus; NS, non-significant; VPN, ventroposterior nuclei. Bold font signifies statistical significance.*

**Table 3:** Semi-quantitative analysis of Iba1 immunofluorescence per region.

		24 h (P9)			7 days (P15)		
Region	Sex	Saline	Poly I:C	*p*-Value	Saline	Poly I:C	*p*-Value
Hippocampus CA1 Median [IQR]	**Male**	370 [57–484]	1021 [801–1125]	**0.008**	527 [344–585]	341 [273–929]	NS
	**Female**	610 [66–617]	1283 [1070–1750]	**0.004**	404 [87–735]	1253 834–1412]	**0.04**
Hippocampus DG Median [IQR]	**Male**	333 [44–538]	1342 [993–1826]	**0.008**	455 [289–518]	296 [243–565]	NS
	**Female**	260 [52–479]	1748 [1601–2505]	**0.004**	285 [63–511]	1087 [597–1370]	**0.01**
Thalamus VPN Median [IQR]	**Male**	305 [14–388]	635 [434–734]	**0.03**	417 [250–519]	255 [219–365]	NS
	**Female**	254 [32–279]	1039 [773–1188]	**0.008**	187 [41–390]	824 [426–1059]	**0.04**
Cingulate Cortex Median [IQR]	**Male**	314 [70–458]	903 [728–988]	**0.009**	298 [239–390]	279 [250–482]	NS
	**Female**	249 [93–397]	899 [741–980]	**0.004**	284 [107–609]	830 [525–1186]	NS

*Data show immunofluorescence arbitrary units (millions). *n* = 5–6/group. Mann–Whitney *U*-test used for analysis. DG, dentate gyrus; NS, non-significant; VPN, ventroposterior nuclei. Bold font signifies statistical significance.*

### CHOP Expression Following Poly I:C

**FIGURE 5:**
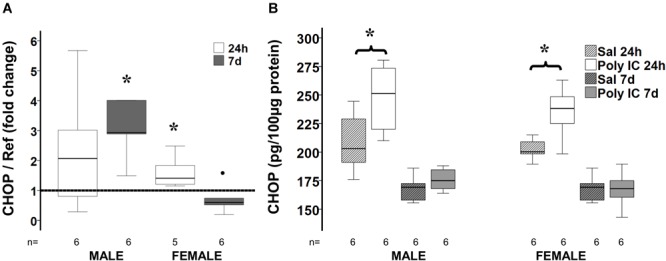
Delayed sex-specific CHOP expression after poly I:C in P8 mice. Fold change in CHOP gene **(A)** and protein **(B)** expression at 24 h and 7 days after poly I:C exposure at P8. Data are shown using box and whiskers plots, where boxes are limited by the 75^th^ and 25^th^ percentiles (IQR) and whiskers are limited by the last data point within 1.5 times the IQR from the median (continuous line inside the box). Fold change was calculated relative to NS-treated mice shown as discontinuous line sitting at 1). °, outlier (between 1.5 and 3 times the IQR); ^•^, extreme (>3 times the IQR); ^∗^*p* < 0.05.

Both CHOP gene and protein expression were increased 24 h after poly I:C exposure in males and female mice (Figure 5). However, only in the male mice, 7 days after poly I:C sexual dimorphism in CHOP gene expression resulted in a threefold increase compared to NS treated mice suggesting persistent ER stress in male mice. Expression of GRP94, an important ER chaperone for TLR3 and a known protective factor against ER stress, was not different between treatment groups in either sex.

The full dataset analyzed and reported in the result section is available to the reader as Supplementary Material.

## Discussion

Our pilot results suggest that TLR3 activation affects the developing brain differently in male and female mice. Early caspase activation is more prominent in the brain of female mice 6 h after exposure to poly I:C at P8. Here, we also document sexually specific temporal patterns of pro-inflammatory cytokine/chemokine expression. Early IRF-3 nuclear translocation downstream of TLR3 activation leads to INF-β upregulation 6 h after poly I:C in female mice, while delayed NFκB-IRF-3 nuclear translocation leads to INF-β upregulation 14 h after exposure in male mice. Although, several pro-inflammatory cytokines such as IL-1β, CXCL-10, TNF-α, MCP-1, FAS, and IL-6 are increased by 6 h after poly I:C in both sexes, some of them, IL-6 and FAS, remain upregulated at 14 h after poly I:C in conjunction with IL-10 downregulation only in the male mice. Earlier activation of astrocytes after poly I:C may explain the increased pro-inflammatory profile seen in male mice at 14 h after exposure. While most of the pro-inflammatory markers return to levels similar to those seen in NS-treated mice sometime between 24 h and 7 days after poly I:C in male mice; late increase in INF-γ, CXCL-1, and CXCL-10, combined with decrease IL-10, and persistent microglia activation suggest a greater late pro-inflammatory state in female mice 7 days after poly I:C. We speculate that a second “wave” of INF-β upregulation documented in male mice 7 days after poly I:C, may prevent release of INF-γ, microglia activation, and downstream release of CXCL-10 in the male mice (Ottum et al., 2015; Kovarik et al., 2016). Mechanistically, delayed CHOP upregulation in male mice 7 days after poly I:C may prevent cytokine translation in male mice (Moon, 2014). Altogether, TLR3 activation produces early activation of apoptotic pathways predominantly in female mice, early pro-inflammatory cytokine profile in both sexes, which extends at least for 7 days after poly I:C exposure in female mice presumptively secondary to persistent microglia activation. The role of ER-stress in the sexual dimorphism in response to poly I:C is still unclear.

Sex differences in the mechanisms of cell death have been described after brain injury secondary to stroke (Liu et al., 2011) and hypoxia-ischemia (Hagberg et al., 2004; Zhu et al., 2007; Northington et al., 2011; Chavez-Valdez et al., 2012) and here, we provide preliminary evidence that these differences may also occur in response to TLR3 activation in the neonatal brain. In the developing brain, a greater acute proclivity to apoptotic cell death in females and to a pro-inflammatory necrotic-like cell death in males has been documented (Hurn et al., 2005; Vannucci and Hurn, 2009; Northington et al., 2011). Cultured female neurons are more sensitive to etoposide-induced apoptosis, while cultured male neurons are more sensitive to excitotoxic stress (Du et al., 2004). Accordingly, following perinatal hypoxia-ischemia or ischemic brain injury, inhibition of caspases provides protection to female rodents (Renolleau et al., 2007), while inhibition of PARP-1-mediated necrotic pathways provides protection to male rodents (Hagberg et al., 2004). Activation of TLR3 by poly I:C leads to both types of cell death. TLR3-mediated apoptotic cell death occurs via upregulation of death-receptors 4/5 and a transactivating p63 isoform α-mediated and IRF3-mediated initiation of the extrinsic pathway (Sun et al., 2011; Gambara et al., 2015) or via a death receptor devoted RIP1-mediated TLR3/caspase-8 complex formation (Estornes et al., 2012). On the other hand, TLR3-mediated necrotic-like cell death, such as programmed necrosis, occurs in the setting of caspase-8 inhibition via a RIP3 kinase-dependent mechanism that involves TRIF and MLKL, but independent of RIP1 (He et al., 2011; Kaiser et al., 2013; Lotzerich et al., 2018). Indirectly, TNF death receptor activation following exposure to TLR3 ligands may proceed via classic RIP1-RIP3 necroptosis pathway, which is also prominent in male mice in the P7 model of hypoxia-ischemia (Northington et al., 2011; Kaiser et al., 2013; Chavez-Valdez et al., 2014). Our results suggest that similar to stroke and hypoxia-ischemia, the mechanisms of cell death in the developing brain after TLR3 activation diverge by sex. The greater caspase 3 and caspase 8 activity documented in the brain of female mice 6 h after poly I:C suggest a preferential activation of the apoptotic extrinsic pathway compared to male mice. Furthermore, IRF3 mediated initiation of apoptotic extrinsic pathway is suggested by the increased IRF3 nuclear translocation demonstrated 6 h after poly I:C in female mice, and event that coincided with caspase activation.

There is also evidence for intrinsic sex-specific differences in inflammatory responses induced by activation of immune cells in the immature brain (Mallard et al., 2018). Additionally, peripheral inflammatory responses may also have an influence in severity of brain injury in a sex-specific manner. For example, peripheral depletion of circulating myeloid cells reduces brain inflammation and injury in male but not female mice (Smith et al., 2018). Further, in models of non-viral septic peritonitis and ischemic bowel injury, TLR3 genetic deletion attenuates the perpetuation of cytokines and chemokines release by macrophages, recruitment of neutrophils, systemic inflammation, and multiorgan injury (Cavassani et al., 2008). Similar role of TLR3 activation as an amplifier of the early inflammatory responses have been reported in the kidney and the liver (Patole et al., 2005; Lang et al., 2006). Activation of TLR3 with poly I:C induces a prolonged inflammatory response from cultured astrocytes (Chistyakov et al., 2018), microglia (Dupuis et al., 2016; Yousif et al., 2018), and brain endothelial cells (Johnson et al., 2018). The prolonged inflammatory response induced by TLR3 activation in microglia and astrocytes biologically serves to control or to enhance insidious viral infections such as Chikungunya (Priya et al., 2014) and HIV-1 (Bhargavan and Kanmogne, 2018) and non-viral infections such as Borrelia burgdorferi (Greenmyer et al., 2018). Sex differences in inflammatory responses depend on the specific intracellular TLR ligand used. Male peripheral mononuclear cells (PMNCs) treated with TLR7 ligands produce less INF than females cells, while male PMNCs treated with TLR8 and TLR9 agonists produce more IL-10 than female cells (Torcia et al., 2012). Thus, the activation of TLR7, 8, and 9 may provide a net anti-inflammatory effect in the males. However, the role of sex on the TLR3-mediated prolonged inflammatory response is unknown in the developing brain until now. Our experiments suggest persistent increase in pro-inflammatory cytokines IL-6, FAS and INF-β, and decrease in IL-10 in the brain of male mice up to 14 h after poly I:C, which contrast with the response in the brain of female mice. We speculate that this is the result of more acute/subacute pro-inflammatory necrotic type of cell death with earlier astrocytic activation in male mice. The pro-inflammatory response appears to resolve in the male mice sometime between 24 h and 7 days after poly I:C. In contrast, a pro-inflammatory profile persist at least until 7 days after poly I:C in female mice. The expression of INF-β after poly I:C may explain the lack of INF-γ expression in both males and female pups up to 24 h (Ottum et al., 2015). In contrast, the lack of INF-β upregulation documented in female mice 7 days after poly I:C, may explain the late upregulation of INF-γ, microglia activation and downstream CXCL-10 expression (Kovarik et al., 2016). However, the role of systemic inflammation in the persistent pro-inflammatory profile documented in the brain of female mice after poly I:C is still unclear.

We acknowledge limitations in this study. We have not dissect the few discrepancies between transcription and translation of certain markers, such as IL-1β or CHOP at 24 h, which shows sex dimorphism in gene expression. Further, we have not studied the influence of systemic inflammation in the brain. Knowing that the blood–brain barrier (BBB) integrity is altered by systemic inflammation, disrupting immune cell trafficking to the brain and leading to activation of astrocytes within the BBB (Banks, 2005; Abbott et al., 2006); our exposure paradigm using IP poly I:C will presumably induce both a primary inflammatory response, as well as a systemic-derived response in the brain secondary to glial activation by circulating cytokines released during TLR3 activation of cells in other organs (e.g., macrophages, adipocytes, keratinocytes). Although the complex interactions between systemic and brain inflammatory responses cannot be further elaborated with the reported set of experiments, TLR3 activation by poly I:C appears to produce only a modest systemic inflammatory short lasting response compared to responses to LPS (Packard et al., 2012), thus we speculate that the earlier GFAP peak at 14 h after poly I:C, may be the result of a greater influence of systemic inflammation in the brain of male mice as previously reported (Smith et al., 2018), which may also explain the increase IL-6, FAS and decrease IL-10 seen simultaneously in males. Similarly, we cannot conclude about the cellular source of the sexual dimorphism in cytokine expression documented in female mice 7 days after poly I:C exposure. However, the simultaneous lack of INF-β gene upregulation, increase INF-γ levels, increase Iba-1 expression across brain regions, and increase CXCL-10 levels, allow us to speculate that persistently activated microglia may be the source of these changes. Other mechanisms by which poly I:C may also produce effects in the brain (e.g., activation of inflammasome) as suggested by others (Rajan et al., 2010; Franchi et al., 2014) need further studies. In rodents, IP injection of poly I:C at less than half of the dose used in our experiments (3–4 instead of 10 mg/kg) produces fever within 5 to 14 h after injection (Hopwood et al., 2009) and also impairs endothelial function (Zimmer et al., 2011). Since a decrease in core temperature may suppress microglial induction and attenuate inflammation, the evaluation of temperature changes in response to poly I:C also needs further investigation. Finally, our experiments are not powered to study differences within sexes and the analysis has been limited to differences between treatments within each sex group. Thus, larger studies, with 20–30 mice per group depending on the primary outcomes used for power calculation, are needed to confirm our preliminary observations between sexes.

Perinatal TLR3 activation is speculated as a mechanism leading to several neurodevelopmental, neurodegenerative, and neuropsychiatric disorders (Brown, 2006; De Miranda et al., 2010; Arroyo et al., 2011; Forrest et al., 2012; Kneeland and Fatemi, 2013). The male predominance in these disorders (McGrath, 2006; Loomes et al., 2017), matches the early sex differences documented within the first 24 h after TLR3 activation in our experiments. We propose that following exposure of the developing brain to TLR3 ligand, female cells acutely die via apoptosis, while male cells die via necrotic-like cell death (i.e., programmed necrosis), as suggested in other models of neonatal brain injury (Hagberg et al., 2004; Zhu et al., 2007; Northington et al., 2011; Chavez-Valdez et al., 2012). The early release of DAMPs from necrotic cells and the effects of systemic inflammation may intensify astrocytosis in male mice, with downstream nuclear translocation of transcription factors and production of pro-inflammatory mediators within the first 24 h. The role of the late pro-inflammatory response documented in female mice after TLR3 activation in the development of neurological disorders is unclear. However, emergent literature is suggesting a protective role of inflammatory mediators against neurodegenerative diseases (Lemere, 2007; Ottum et al., 2015; Le et al., 2016). Further experiments better powered to confirm our speculations are needed to then study the role of these potential sex differences in the sex dimorphism seen in many of neurological disorders linked to TLR3 activation early in life.

## Ethics Statement

We confirm that any aspect of the work covered in this manuscript involving experimental animals has been conducted with the ethical approval of all relevant bodies.

## Author Contributions

RC-V, FN, and CM: experimental design. AM and LS: animal works. RC-V, AM, LS, TY, and LJ: tissue processing. RC-V, TY, and LJ: immunohistochemistry, PCR, and biochemical assays. RC-V: statistical analysis and initial draft preparation. All authors: critical reviews of the manuscript and approval of final version.

## Conflict of Interest Statement

The authors declare that the research was conducted in the absence of any commercial or financial relationships that could be construed as a potential conflict of interest.

## Supplementary Material

TABLE S1 Manuscript’s dataset.
